# A lake classification concept for a more accurate global estimate of the dissolved inorganic carbon export from terrestrial ecosystems to inland waters

**DOI:** 10.1007/s00114-018-1547-z

**Published:** 2018-03-26

**Authors:** Fabian Engel, Kaitlin J. Farrell, Ian M. McCullough, Facundo Scordo, Blaize A. Denfeld, Hilary A. Dugan, Elvira de Eyto, Paul C. Hanson, Ryan P. McClure, Peeter Nõges, Tiina Nõges, Elizabeth Ryder, Kathleen C. Weathers, Gesa A. Weyhenmeyer

**Affiliations:** 1 Department of Ecology and Genetics/Limnology, Uppsala University, Norbyvägen 18D, 752 36 Uppsala, Sweden; 2 Odum School of Ecology, University of Georgia, Athens, GA 30602 USA; 3 Department of Biological Sciences, Virginia Tech, Derring Hall, Blacksburg, VA 24061 USA; 4 Bren School of Environmental Science and Management, University of California, Santa Barbara, CA 93106 USA; 5 Instituto Argentino de Oceanografía (UNS-CONICET), Florida 8000 (Camino La Carrindanga km 7,5), B8000BFW Bahía Blanca, Buenos Aires Argentina; 6 Department of Ecology and Environmental Sciences, Umeå University, Linnaeus väg 6, 901 87 Umeå, Sweden; 7 Center for Limnology, University of Wisconsin-Madison, 680 N. Park St., Madison, WI USA; 8 Marine Institute, Furnace, Newport, Co. Mayo Ireland; 9 Centre for Limnology, Estonian University of Life Sciences, Kreutzwaldi 1, 51014 Tartu, Estonia; 10 Centre for Freshwater and Environmental Studies, Dundalk Institute of Technology, Dundalk, Co Louth Ireland; 11 Cary Institute of Ecosystem Studies, Millbrook, NY 12545 USA

**Keywords:** Global carbon cycle, Lake functioning, Hydrologic CO_2_ transport, Lake carbon cycling, Earth system models, Lake primary production

## Abstract

The magnitude of lateral dissolved inorganic carbon (DIC) export from terrestrial ecosystems to inland waters strongly influences the estimate of the global terrestrial carbon dioxide (CO_2_) sink. At present, no reliable number of this export is available, and the few studies estimating the lateral DIC export assume that all lakes on Earth function similarly. However, lakes can function along a continuum from passive carbon transporters (passive open channels) to highly active carbon transformers with efficient in-lake CO_2_ production and loss. We developed and applied a conceptual model to demonstrate how the assumed function of lakes in carbon cycling can affect calculations of the global lateral DIC export from terrestrial ecosystems to inland waters. Using global data on in-lake CO_2_ production by mineralization as well as CO_2_ loss by emission, primary production, and carbonate precipitation in lakes, we estimated that the global lateral DIC export can lie within the range of ${0.70}_{-0.31}^{+0.27}$ to ${1.52}_{-0.90}^{+1.09}$ Pg C yr^−1^ depending on the assumed function of lakes. Thus, the considered lake function has a large effect on the calculated lateral DIC export from terrestrial ecosystems to inland waters. We conclude that more robust estimates of CO_2_ sinks and sources will require the classification of lakes into their predominant function. This functional lake classification concept becomes particularly important for the estimation of future CO_2_ sinks and sources, since in-lake carbon transformation is predicted to be altered with climate change.

## Integrating inland waters into Earth system models

Earth system models (ESMs) simulate the interactions between global climate and biogeochemical cycles based on the physical, chemical, and biological properties of the three main components of the Earth system: land, atmosphere, and ocean. Connecting atmospheric transport, ocean circulation, and terrestrial biosphere models (TBMs; land) allows simulation of carbon stores and fluxes at the global scale (Falkowski et al. 2000; IPCC 2013). TBMs simulate biogeochemical and physical processes of terrestrial ecosystems, including up to 25 key processes (Fisher et al. 2014). While global terrestrial gross primary production (GPP_land_) can be measured using satellite remote sensing data, autotrophic and heterotrophic respiration of land ecosystems (R_a_ and R_h_, respectively) need to be simulated in current TBMs. In TBMs, the biomass production on land is quantified as net primary production (NPP_land_; NPP_land_ = GPP_land_ − R_a_), while the amount of carbon stored in or released from terrestrial ecosystems, terrestrial net ecosystem production (NEP_land_), is obtained by subtracting the total terrestrial ecosystem respiration (R_a_ + R_h_) from GPP_land_ (Fisher et al. 2014). Inland waters connect the Earth system components land and ocean (Cole et al. 2007; Drake et al. 2017; Tranvik et al. 2009). However, current ESMs do not simulate carbon fluxes in inland waters (Bauer et al. 2013). Instead, inland waters were for a long time regarded as “passive pipes” between land and ocean. Recently, aquatic carbon fluxes were integrated into TBMs on a regional scale (Langerwisch et al. 2016).

In recent years, the view of inland waters being passive carbon transporters between land and ocean has changed. Instead, lakes have been identified as important regulators of carbon processing along the land to ocean aquatic continuum (LOAC) (Battin et al. 2009; Biddanda 2017; Cole et al. 2007; Tranvik et al. 2009). The functioning of lakes in carbon transport and transformation controls both lateral (i.e., hydrologic transport) and vertical (i.e., emission and burial) carbon fluxes along the LOAC and thus influences the global carbon balance (Battin et al. 2009; Biddanda 2017; Cole et al. 2007; Tranvik et al. 2009). These findings have important implications for the calculation of the NEP_land_ in TBMs. When the proportion of R_h_ from terrestrial biomass that leaves terrestrial ecosystems through lateral hydrologic export to streams and lakes (Oquist et al. 2014) is not accounted for when simulating R_h_, NEP_land_ is overestimated. The recognition of the importance of lateral aquatic carbon transport for continental carbon budgets has led to the realization that terrestrial ecosystems are less efficient in sequestering carbon than previously assumed (Butman et al. 2016; Ciais et al. 2008). A recent study showed that the NEP_land_ of the conterminous USA might have been overestimated by more than 25%, as lateral aquatic carbon fluxes were not accounted for in present TBMs (Butman et al. 2016). Thus, realistic estimates of the terrestrial carbon sink/source require accurate quantification of the lateral carbon export from terrestrial ecosystems to inland waters. These estimates are currently not available and are unrealistic to measure over large geographic regions. Thus, the inclusion of lateral inland water carbon fluxes into global ESMs remains difficult, but essential if we seek to reconcile global carbon budgets (Battin et al. 2009; Butman et al. 2016; Weyhenmeyer et al. 2015).

## Lake functioning along the aquatic continuum

**Fig. 1:**
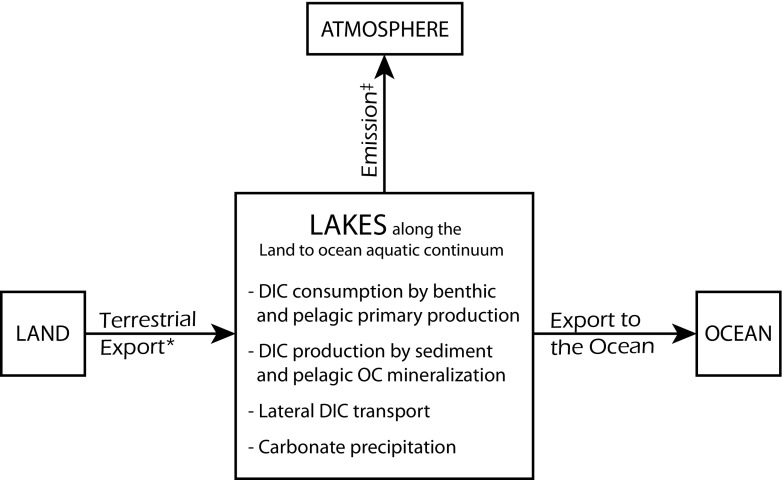
Schematic representation of the role of lakes in dissolved inorganic carbon (DIC) cycling along the land to ocean aquatic continuum (LOAC) showing the main global DIC fluxes and in-lake transformation processes. Organic carbon sedimentation and burial is not included in this conceptualization, since we restricted our analysis to DIC fluxes along the LOAC. *Includes surface and groundwater transport from land to lakes. ^‡^In lakes with high DIC consumption, uptake of atmospheric CO_2_ can partly exceed total emissions resulting in temporarily negative net emissions

Rivers, floodplains, and lakes control carbon transport as well as transformation along the LOAC (Cole et al. 2007; Raymond et al. 2013; Tranvik et al. 2009). The integration of lakes into global carbon dioxide (CO_2_) budgets is difficult, since lakes function differently depending on their characteristics and location (Tranvik et al. 2009). Nutrient conditions, hydrology, catchment characteristics, lake morphology, and regional climate are important factors determining the functioning of lakes in the global carbon cycle (Lewis Jr. 2011; Tranvik et al. 2009; Weyhenmeyer et al. 2015). The role of lakes in dissolved inorganic carbon (DIC) transport and transformation (Fig. 1) depends on the characteristics of each lake. In-lake CO_2_ consumption and production might, for example, be the most important drivers of lake carbon dynamics in warm eutrophic lakes (Almeida et al. 2016), while lateral CO_2_ transport can be highly significant in boreal lakes (Weyhenmeyer et al. 2015). Although decomposition rates of organic carbon are higher in lakes with short water residence times (Catalán et al. 2016), short water residence times generally result in lower in-lake CO_2_ production and consumption, if the majority of carbon is transported downstream before being processed (Tranvik et al. 2009).

At present, different assumptions regarding the functioning of lakes in the global carbon cycle are found in the literature. Many global carbon estimates, including those currently used for policy decisions (IPCC 2013), assume that in lakes, CO_2_ is efficiently produced by mineralization of terrestrial dissolved organic carbon (DOC) (Aufdenkampe et al. 2011; Battin et al. 2009; Cole et al. 2007; IPCC 2013; Raymond et al. 2013; Tranvik et al. 2009). However, assuming that all lakes function similarly can be problematic since the transformation of DOC to CO_2_ in many lakes is less efficient than previously thought (McDonald et al. 2013; Stets et al. 2009; Weyhenmeyer et al. 2015). In numerous lakes, a large proportion of the emitted CO_2_ originates from terrestrial ecosystem respiration (R_h_) and has been transported to inland waters via discharge (Weyhenmeyer et al. 2015). Assuming this CO_2_ to be produced by in-lake DOC mineralization results in an overestimation of NEP_land_ in TBMs and ESMs. Thus, assumptions about lake functioning have a large impact on the calculated lateral DIC export from terrestrial ecosystems to inland waters.

The aim of this study was to develop a conceptual model to quantify variations in the estimate of the global lateral DIC export from terrestrial ecosystems to inland waters, depending on the assumed predominant function of lakes.

## A lake classification concept and effects on the global terrestrial DIC export

**Fig. 2:**
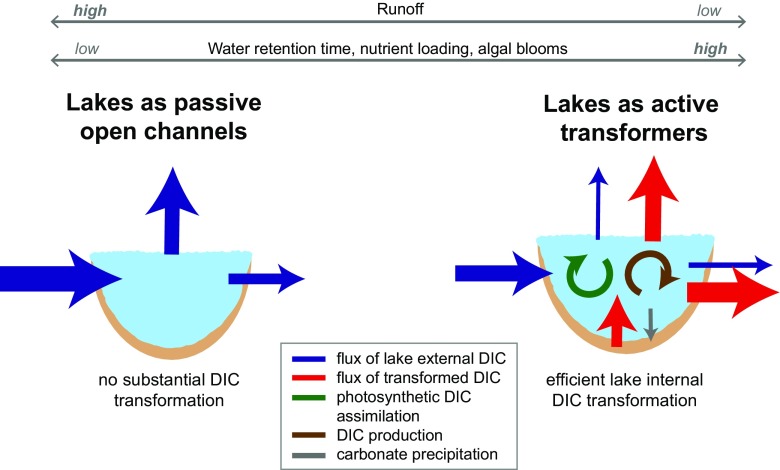
Conceptual figure showing two ends of a continuum of lake functions in the cycling of dissolved inorganic carbon (DIC). The figure also demonstrates how lake functioning may shift depending on runoff, water retention time, nutrient loading, and algal blooms. Organic carbon sedimentation and burial are not included in this conceptualization, since we restricted our analysis to DIC fluxes in lakes

To integrate lakes into ESMs, we classified lakes into three functional categories: (1) lakes as active carbon transformers, (2) lakes as intermediate active carbon transformers, and (3) lakes as passive open channels, where classes 1 and 3 represent the ends of a continuum of possible lake functioning depending on lake characteristics (Fig. 2).

To demonstrate the effect of different assumptions about lake functioning on calculated estimates of DIC export from terrestrial ecosystems to inland waters (streams, rivers, lakes, and reservoirs), we established the following mass-balance equation by accounting for all main DIC fluxes along the LOAC (Fig. 1):

1 $$ {\mathrm{DIC}}_{\mathrm{export}\kern0.5em }={\mathrm{DIC}}_{\mathrm{ocean}}+{\mathrm{CO}}_2\_{\mathrm{emission}}_{\mathrm{lake}}+{\mathrm{GPP}}_{\mathrm{lake}}+{\mathrm{CCP}}_{\mathrm{lake}}\hbox{--} {\operatorname{MIN}}_{\mathrm{lake}}+{\mathrm{CO}}_2\_{\mathrm{emission}}_{\mathrm{lotic}}+{\mathrm{GPP}}_{\mathrm{lotic}}+{\mathrm{CCP}}_{\mathrm{lotic}}\hbox{--} {\operatorname{MIN}}_{\mathrm{lotic}} $$

where DIC_export_ is the DIC exported from terrestrial ecosystems to inland waters, DIC_ocean_ is the DIC entering the oceans via lateral surface and groundwater transport, CO_2__emission_lake_ is the net CO_2_ emission from lakes and reservoirs (hereafter lake), CO_2__emission_lotic_ is the net CO_2_ emission from streams and rivers (lotic systems), GPP_lake_ is the CO_2_ consumption by lake primary production, GPP_lotic_ is the CO_2_ consumption by primary production in streams and rivers, CCP_lake_ is the in-lake calcium carbonate precipitation, CCP_lotic_ is the calcium carbonate precipitation in streams and rivers, MIN_lake_ is the amount of CO_2_ produced by lake mineralization, and MIN_lotic_ is the amount of CO_2_ produced by stream and river mineralization. Organic carbon sedimentation and burial is not included in our conceptual model, since we restricted the analysis to DIC fluxes along the LOAC. Our model (Fig. 2) is meant to provide a conceptual framework that can be applied at different spatial scales with any of the most comprehensive available estimates describing the fluxes stated in Eq. 1.

In lakes that predominantly function as active carbon transformers (e.g., warm eutrophic lake ecosystems (Almeida et al. 2016)), GPP_lake_ is substantial for the lake carbon budget. The terrestrial DIC export for landscapes in which lakes predominantly function as active carbon transformers can be estimated using Eq. 1.

When assuming lakes to function predominantly as intermediate active carbon transformers, CO_2_ emissions from lakes would mainly be sustained by in-lake DOC mineralization, and GPP_lake_ as well as CCP_lake_ would be close to zero. In that case, DIC_export_ can be estimated as:

2 $$ {\mathrm{DIC}}_{\mathrm{export}\kern0.5em }={\mathrm{DIC}}_{\mathrm{ocean}}+{\mathrm{CO}}_2\_{\mathrm{emission}}_{\mathrm{lotic}}+{\mathrm{GPP}}_{\mathrm{lotic}}+{\mathrm{CCP}}_{\mathrm{lotic}}\hbox{--} {\operatorname{MIN}}_{\mathrm{lotic}} $$

This intermediate lake function reflects that some lakes, often referred to as heterotrophic lakes, have high bacterial and photochemical CO_2_ production, and DOC-derived CO_2_ is the main source of lake CO_2_ emissions. These lakes have little phytoplankton and zooplankton production, which is typical for some boreal lakes (Jonsson et al. 2001) and probably even for nutrient-poor and deep lakes. Assuming CO_2__emission_lake_ and MIN_lake_ approach zero in Eq. 2 does not indicate that these lakes have no in-lake carbon transformation, but that the CO_2_ production in these lakes is mainly sustained by mineralization of allochthonous organic carbon. Thus, for this lake type, CO_2__emission_lake_ cannot be included as flux term when calculating the DIC export from terrestrial ecosystems to inland waters. This lake function reflects the way global lakes are currently accounted for in ESMs, where it is assumed that CO_2_ in inland waters originates mainly from in-lake mineralization of allochthonous organic carbon (Aufdenkampe et al. 2011; Battin et al. 2009; Cole et al. 2007; IPCC 2013; Raymond et al. 2013; Tranvik et al. 2009).

When instead assuming that lakes predominantly function as passive open channels (e.g., small boreal and temperate lakes (Jonsson et al. 2003; Stets et al. 2009)), GPP_lake_, CCP_lake_, and MIN_lake_ are minor, and CO_2_ emissions are mainly sustained by hydrologic DIC inflow to lakes that is derived from terrestrial inorganic carbon export. In this case, we assumed GPP_lake_, CCP_lake_, and MIN_lake_ to approach zero in Eq. 1. Thus, with lakes as passive open channels, the DIC_export_ can be estimated as:

3 $$ {\mathrm{DIC}}_{\mathrm{export}\kern0.5em }=\kern0.5em {\mathrm{DIC}}_{\mathrm{ocean}}+{\mathrm{CO}}_2\_{\mathrm{emission}}_{\mathrm{lake}}+{\mathrm{CO}}_2\_{\mathrm{emission}}_{\mathrm{lotic}}+{\mathrm{GPP}}_{\mathrm{lotic}}+{\mathrm{CCP}}_{\mathrm{lotic}}\hbox{--} {\operatorname{MIN}}_{\mathrm{lotic}} $$

## Sensitivity of the global terrestrial DIC export to lake functioning

To demonstrate the sensitivity of the global DIC_export_ to the assumed functioning of global lakes using Eqs. 1, 2, and 3, we collected published data on global DIC_ocean_, CO_2__emission_lake_, CO_2__emission_lotic_, GPP_lake_, MIN_lake_, and MIN_lotic_ from the literature (Table 1). For our calculations, we chose the most recent estimate of each respective flux, since these were the most accurate available flux estimates on the global scale. While the estimate for GPP_lake_ from Lewis Jr. (2011) used in Eq. 1 already entails a scaling of lake GPP based on the latitudinal distribution of lakes and prevailing nutrient conditions, Raymond et al. (2013) simulated CO_2__emission_lake_ from non-tropical lakes using DOC and lake area and used a median value for tropical lakes in their estimate. Thus, the Raymond et al. (2013) model does not account for CO_2_ emissions derived from lateral DIC inputs to lakes; hence, lakes that function as passive open channels are excluded. Consequently, scaling global CO_2__emission_lake_ and MIN_lake_ according to the assumed predominant lake functions (Eqs. 1, 2, and 3) is reasonable.

**Table 1:** Overview of global estimates for dissolved inorganic carbon (DIC) fluxes in inland waters. Values used for the calculation of the DIC export from terrestrial ecosystems to inland waters in our study, i.e., the most recent estimates for the respective flux, are in bold. The upper and lower limits indicate the range of the estimates reported in the respective studies

Flux	Abbreviation	Flux estimate [Pg C yr^−1^]	Reference
DIC export to the oceans	DIC_ocean_	${\mathbf{0.45}}_{-\mathbf{0.11}}^{+\mathbf{0.10}}$	(Cole et al. 2007)
CO_2_ emissions from streams and rivers	CO_2__emission_lotic_	${\mathbf{0.65}}_{-\mathbf{0.20}}^{+\mathbf{0.17}}$	(Lauerwald et al. 2015)
		${1.8}_{-0.25}^{+0.25}$	(Raymond et al. 2013)
		0.56	(Aufdenkampe et al. 2011)
CO_2_ emissions from lakes and reservoirs	CO_2__emission_lake_	${\mathbf{0.32}}_{-\mathbf{0.26}}^{+\mathbf{0.52}}$	(Raymond et al. 2013)
		0.64	(Aufdenkampe et al. 2011)
CO_2_ consumption by lake gross primary production	GPP_lake_	${\mathbf{1.3}}_{-\mathbf{0.25}}^{+\mathbf{0.21}}$	(Lewis Jr. 2011)
		0.65	(Pace and Prairie 2005)
Lake calcium carbonate precipitation	CCP_lake_	**0.03**	(Meybeck 1993)
CO_2_ production by in-lake mineralization	MIN_lake_	${\mathbf{0.83}}_{-\mathbf{0.08}}^{+\mathbf{0.09}}$	(Pace and Prairie 2005)
CO_2_ production by mineralization in rivers	MIN_lotic_	**0.40**	(Caraco and Cole 1999)

**Fig. 3:**
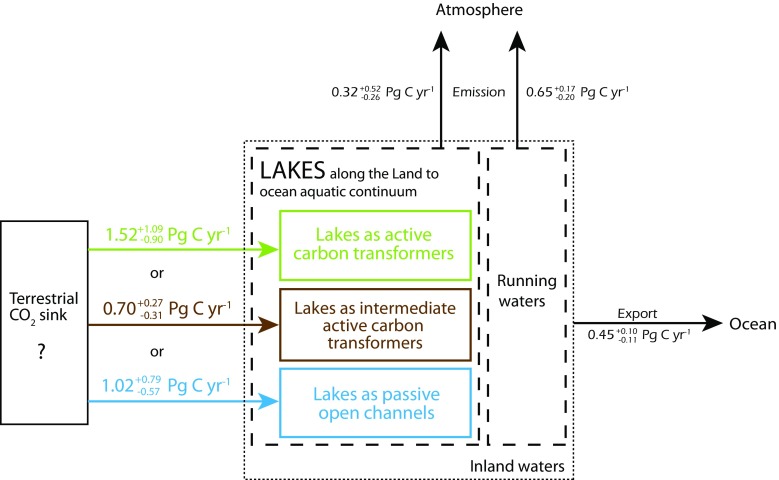
Effect of considered functioning of global lakes on estimates of the lateral dissolved inorganic carbon (DIC) export from terrestrial ecosystems to inland waters in relation to CO_2_ transport from inland waters to the atmosphere and the ocean. Depending on the considered functioning of global lakes, the calculated lateral DIC export from terrestrial ecosystems to inland waters varies between ${0.70}_{-0.31}^{+0.27}$ and ${1.52}_{-0.90}^{+1.09}$ Pg C yr^−1^. The DIC transformation and CO_2_ emission in running waters are kept constant for all cases. Numbers in black font from Cole et al. (2007), Raymond et al. (2013), and Lauerwald et al. (2015)

Our sensitivity analysis illustrates the likely range of the calculated DIC export from terrestrial ecosystems to inland waters under the assumption of different lake functions. When assuming that all lakes on Earth function as active carbon transformers, DIC_export_ according to Eq. 1 equaled ${1.52}_{-0.90}^{+1.09}$ Pg C yr^−1^. DIC_export_ became smallest, i.e., ${0.70}_{-0.31}^{+0.27}$ Pg C yr^−1^, when we considered lakes as intermediate active carbon transformers (Eq. 2). When we considered lakes as passive open channels (Eq. 3), the DIC_export_ turned to ${1.02}_{-0.57}^{+0.79}$ Pg C yr^−1^. Thus, we found that calculations of DIC_export_ can vary between ${0.70}_{-0.31}^{+0.27}$ and ${1.52}_{-0.90}^{+1.09}$ Pg C yr^−1^, depending on the assumed predominant function of lakes (Fig. 3). These numbers for the lateral DIC export are about 25 and 50% of the total carbon export from terrestrial ecosystems to inland waters estimated by previous studies (Aufdenkampe et al. 2011; Battin et al. 2009; Tranvik et al. 2009). We suggest that the variability in calculated lateral DIC fluxes from terrestrial ecosystems to inland waters has a strong influence on estimates of the terrestrial CO_2_ sink and might explain a share of the residual terrestrial CO_2_ sink of approximately 2 Pg C yr^−1^ (Houghton 2003; Nakayama 2017; Schimel 1995). It should be noted that DIC_export_ comprises DIC originating from rock weathering as well as soil-derived CO_2_. Only about 70% of DIC_export_ might be soil derived (i.e., of atmospheric origin), while the other 30% is derived from rock weathering and is therefore part of the slow carbon cycle and has no atmospheric origin (Ciais et al. 2008; Einsele et al. 2001).

In our analysis, we used the flux estimate of Lauerwald et al. (2015) for CO_2__emission_lotic_, since this study simulates CO_2_ emissions from running waters at a much higher resolution than earlier approaches. To our knowledge, no global estimates for GPP_lotic_ and CCP_lotic_ exist. We set GPP_lotic_ and CCP_lotic_ to zero when testing the sensitivity of DIC_export_ to lake functioning, since the fluxes in streams and rivers were kept constant for all three functional lake classes and thus did not influence the result of the sensitivity analysis. Except for the estimates for GPP_lake_ and MIN_lake_ (Pace and Prairie 2005) as well as MIN_lotic_ (Caraco and Cole 1999), which were indicated in moles C yr^−1^, the values were directly used from the cited publications. The fluxes given in moles C yr^−1^ were converted into fluxes in g C yr^−1^ by multiplying the given values by the atomic mass of carbon. For flux estimates reported as a range in the original study, we used the mid-range for our calculations. The uncertainties presented here indicate the range of the estimates reported in the respective studies (Table 1). The uncertainties of the respective flux estimates used in Eqs. 1, 2, and 3 were summed, resulting in the reported uncertainties of DIC_export_. In our calculations, GPP_lake_ comprises CO_2_ uptake by phytoplankton from all available sources (Lewis Jr. 2011), and MIN_lake_ is a lumped value for the total pelagic and sediment mineralization of autochthonous and allochthonous organic carbon (Pace and Prairie 2005). For a more detailed description of the values used to calculate DIC_export_, consult the respective studies cited in Table 1.

## Sensitivity of terrestrial DIC export to uncertainties in global flux estimates

The lateral DIC export from terrestrial ecosystem to inland waters is currently highly uncertain (Drake et al. 2017), complicating the calculation of global terrestrial carbon sinks/sources (Butman et al. 2016). The different global estimates used to calculate DIC_export_ (Table 1) each entail an uncertainty that sums to the uncertainty of our calculated DIC_export_. Uncertainties in DIC_export_ have previously been estimated as ~ ±1.0 Pg C yr^−1^ (Regnier et al. 2013), which is close to the uncertainty of our calculated DIC_export_ of ${1.52}_{-0.90}^{+1.09}$ (Eq. 1). Although DIC_export_ lies most likely between 0.70 and 1.52 Pg C yr^−1^, the full range of the calculated DIC_export_ when considering the upper and lower boundary value for active carbon transformers and intermediate active carbon transformers is 0.39 to 2.61 Pg C yr^−1^ (Fig. 3).

To demonstrate the relevance of our functional lake classification concept in comparison to the uncertainties of the global flux estimates used in Eqs. 1, 2, and 3, we performed an analysis on the sensitivity of DIC_export_ to uncertainties in the global flux estimates used (Table 1). We varied each respective value used in Eqs. 1, 2, and 3 by ± 25% and compared our calculated DIC_export_ to the DIC_export_ calculated with an error of ± 25% (DIC_export_error_). While the differences in lake functioning resulted in a variation of DIC_export_ of 0.82 Pg C yr^−1^, DIC_export_error_ differed from DIC_export_ by 0.76, 0.35, and 0.51 Pg C yr^−1^ for active carbon transformers, intermediate active carbon transformers, and passive open channels, respectively. Thus, when assuming an error of ± 25% for each respective flux term in Eqs. 1, 2, and 3, the variability in DIC_export_ resulting from different assumptions on lake functioning was larger than the variability between DIC_export_ and DIC_export_error_. These results further highlight the importance of considering lake functioning when calculating DIC_export_.

When testing the sensitivity of DIC_export_ to lake functioning, we set GPP_lotic_ and CCP_lotic_ to zero, since to our knowledge, no global estimates on GPP_lotic_ and CCP_lotic_ exist. To examine the effect of setting GPP_lotic_ (that is definitely > 0) to zero when testing the sensitivity of DIC_export_ to lake functioning, we assumed GPP_lotic_ as 25% of GPP_lake_ and re-calculated DIC_export_. Adding GPP_lotic_ resulted in an increase of DIC_export_ for active carbon transformers (Eq. 1) of 21%. We did not test for the effect of non-zero GPP_lotic_ for the other lake types, since we had assumed GPP_lake_ to approach zero for intermediate active and passive lakes, and GPP_lotic_ is usually considerably smaller than GPP_lake_.

## Spatial variations in lake functioning

Our calculated DIC_export_ estimates were based on the assumption that all lakes on Earth are either active carbon transformers, intermediate active carbon transformers, or passive open channels. This assumption does not depict reality since in some regions on Earth, lakes will rarely function as passive open channels or active carbon transformers. Although it is beyond the scope of this study to allocate a lake function to each of the 117 million lakes on Earth, we made a rough estimate of how many lakes potentially can function as active carbon transformers or as passive open channels. Based on Lewis Jr. (2011), we assumed that all lakes located between 39° N and 39° S have the potential to function as active carbon transformers. We chose this latitude as a borderline for potentially active lakes, since the modeled median lake gross primary production of global lakes increases sharply between 42.5° and 37.5° latitude from ~ 400 to ~ 800 g C m^−2^ yr^−1^ (Lewis Jr. 2011). Accordingly, we assumed that all lakes between 54° N and 84° N may function as passive open channels as these lakes are located in the boreal and subarctic zone, are usually small and shallow, and often function as passive open channels (Weyhenmeyer et al. 2015). Even if not taken into consideration in this estimate, it must be noted that a substantial number of humic-rich boreal lakes most probably function as intermediate active carbon transformers. We did not assign lakes located between 39° and 54° latitude to any of our three categories, since the functioning of lakes within these latitudinal bands might vary strongly depending on nutrient conditions, hydrology, catchment characteristics, lake morphology, and regional climate.

Using the abundance and total area of lakes for 3° latitudinal bands from the Global Water Body database (GLOWABO) (Verpoorter et al. 2014), we found that about 25% of lakes on Earth, corresponding to 35% of the global lake area, potentially function as passive open channels, while 60% of lakes, corresponding to 45% of the global lake area, might act predominantly as active carbon transformers. Since the functioning of different lakes within an ecoregion varies widely (McDonald et al. 2013; Weyhenmeyer et al. 2015), this estimate is a first-order approximation of the global distribution of lake functions according to our conceptual model and can serve as an exemplifying application of our classification concept. This classification according to latitudinal distribution only accounts for the control of climate on lake functioning. Additional factors including hydrologic regime, land-use, and regional geography can exert strong influences on lake functioning and should be considered in future, more accurate estimates of the functioning of lakes in the global carbon cycle.

## Refining terrestrial DIC export estimates

We focused our analysis on the role of lakes as modulators of DIC transport and transformation. It has to be noted that the functioning of streams, rivers, floodplains, and wetlands is also of high importance for DIC transport and transformation along the LOAC (Aufdenkampe et al. 2011; Raymond et al. 2013). At the current stage, the use of our conceptual model for calculating the global lateral DIC export from terrestrial ecosystems to inland waters has limitations, which future studies should address, when more whole-lake carbon budgets and estimates of global carbon fluxes are available. At present, our lake classification concept (Figs. 1 and 2) applies only to open lake systems along the LOAC. While this is the prevalent lake type globally, closed basins can play an important role in arid and semi-arid regions (Einsele et al. 2001; Li et al. 2017). The function of these lakes in the global carbon cycle is presently unknown; however, a recent study estimated that in global closed drainage basins, about 0.15 Pg C yr^−1^ of the incoming DIC is stored (Li et al. 2017). Our estimates are also limited by the lack of data for global GPP_lotic_ and CCP_lotic_. We assumed these fluxes to be zero in Eqs. 1, 2, and 3, but performed a sensitivity analysis to demonstrate the effect of GPP_lotic_ on our DIC_export_ estimate according to Eq. 1. As GPP_lotic_ and CCP_lotic_ are larger than zero, including estimates for GPP_lotic_ and CCP_lotic_ would increase the calculated DIC_export_ rates. Further, the global estimate of CCP_lake_ (Table 1) used in Eq. 1 is relatively low, considering that some lakes, e.g., Attersee and Lake Constance, precipitate 4–17% of the incoming carbonate (Einsele et al. 2001). Thus, we suggest that our estimates for DIC_export_ are conservative, particularly since we used the most recent global estimate for CO_2__emission_lotic_ from Lauerwald et al. (2015) that is substantially lower than earlier estimates of CO_2__emission_lotic_ (Table 1).

A significant amount of uncertainty in our calculated DIC_export_ arises from the variability in lake primary production that complicates quantification of global lake CO_2_ consumption. Lake primary production along a global latitudinal gradient can vary by a factor of 1000 (Jonsson et al. 2003; Melack and Kilham 1974) and significantly differs for individual lakes on decadal scale in relation to climate variation (Pettersson et al. 2003). Depending on the estimates for global lake abundance used, Lewis Jr. (2011) reports a variation of global lake GPP between 1.05 and 1.51 Pg C yr^−1^. This uncertainty of 0.46 Pg C yr^−1^ accounts for a variation in our calculated DIC_export_ for active carbon transformers (1.52 Pg C yr^−1^ acc. to Eq. 1) of about 30%. Neglecting GPP_lake_ in the global DIC budget that considers lakes as active carbon transformers (Eq. 1) would result in a reduction of the calculated DIC_export_ by about 85%. In our calculations, a large share of GPP_lake_ is from GPP in warm, nutrient-rich tropical lakes (Lewis Jr. 2011), which we classified as active carbon transformers. We assumed that all photosynthetically fixed CO_2_ in lakes is of terrestrial origin. This assumption is supported by the fact that a large majority of inland waters is supersaturated with CO_2_ and CO_2_ uptake from the atmosphere is minor in global lakes. However, the relative contribution of terrestrial vs. atmospheric-derived CO_2_ to photosynthetic CO_2_ fixation in inland waters is presently unknown (Drake et al. 2017). Assuming that a share of the photosynthetically fixed CO_2_ is taken up by lakes directly from the atmosphere would lower our estimates of DIC_export_ slightly. In lakes with high hydrologic DIC inputs, primary production can be influenced by terrestrially derived DIC, and CO_2_ emissions in net autotrophic lakes can mainly arise from hydrologic DIC inputs (Stets et al. 2009). Consequently, CO_2_ consumption by primary production in lakes along the LOAC needs to be considered when calculating terrestrial DIC export rates.

The single published estimates for the same global inland water DIC flux differ significantly (Table 1). The accuracy of the global estimates for inland water DIC fluxes partly depends on the resolution at which spatial variabilities are accounted for (Lauerwald et al. 2015; McDonald et al. 2013). The spatial resolution currently used to calculate carbon fluxes along the LOAC of maximum 0.5° is too coarse to account for the diversity and regional distribution of soil types, wetlands, streams, rivers, and lakes (Lauerwald et al. 2015; Regnier et al. 2013). However, attempts to resolve, for example, CO_2_ emissions from the global river network at a higher resolution have progressed constantly during the past 10 years (Lauerwald et al. 2015). With ongoing refinements of global estimates of inland water DIC fluxes, our conceptual model, accounting for the predominant lake functions, can be a valuable tool for more robust estimates of the global DIC export from terrestrial ecosystems to inland waters.

Overall, we show here that the consideration of lake functioning is necessary to estimate the magnitude of the global DIC export from terrestrial ecosystems to inland waters. Similar to our estimations for DIC, we expect that our lake classification concept (Fig. 2) can also be applied to other carbon forms (e.g., DOC) as its transport and transformation processes vary strongly among lakes depending on lake characteristics. Since lake functioning differs widely across the globe (Lewis Jr. 2011; Tranvik et al. 2009), accurate estimates of the lateral DIC export will require the prediction of lake functioning for each of the 117 million lakes on Earth (McDonald et al. 2013; Verpoorter et al. 2014). With constant changes in anthropogenic carbon outputs altering the global carbon cycle (Regnier et al. 2013), and disturbances of natural conditions causing eutrophication or a global temperature rise, lake functioning is likely to change (Gudasz et al. 2010; Lewis Jr. 2011; Fig. 2). The continuously high activity in dam construction on global scale (Zarfl et al. 2015) will probably shift numerous riverine systems towards a state at which they predominantly act as active carbon transformers. Global climate change will likely increase the activity of already existing lakes and reservoirs in carbon transformation, especially in the temperate and boreal region (Flanagan et al. 2003; Gudasz et al. 2010; Tranvik et al. 2009). Thus, our functional lake classification concept becomes particularly important for the calculation of the future lateral DIC export from soils to inland waters and future estimations of terrestrial CO_2_ sinks and sources.
